# Design Rule for Constructing Buckling-Free Polymeric Stencil with Microdot Apertures

**DOI:** 10.3390/polym13244361

**Published:** 2021-12-13

**Authors:** Minju Kim, Jinwon Lee, Junsoo Kim, Segeun Jang, Sang Moon Kim

**Affiliations:** 1 Department of Mechanical Engineering, Incheon National University, Incheon 22012, Korea; 202121050@inu.ac.kr (M.K.); iljwl92@gmail.com (J.L.); 2 John A. Paulson School of Engineering and Applied Science, Harvard University, Cambridge, MA 02138, USA; junsookim@g.harvard.edu; 3 School of Mechanical Engineering, Kookmin University, Seoul 02707, Korea; sjang@kookmin.ac.kr

**Keywords:** structural stability, buckling, UV curable polymer, membranes, aperture

## Abstract

A polymeric stencil with microdot apertures made by using polydimethylsiloxane (PDMS) molds with pillar patterns has many advantages, including conformal contact, easy processability, flexibility, and low cost compared to conventional silicon-based membranes. However, due to the inherent deformability of PDMS materials in response to external pressure, it is challenging to construct structurally stable stencils with high structural fidelity. Here, we propose a design rule on the buckling pressure for constructing polymeric stencils without process failure. To investigate the critical buckling pressure (*P_cr_*), stencils are fabricated by using different PDMS molds with aspect ratio variations (AR: 1.6, 2.0, 4.0, and 5.3). By observing the buckled morphology of apertures, the structures can be classified into two groups: low (AR 1.6 and 2.0) and high (AR 4.0 and 5.3) AR groups, and *P_cr_* decreases as AR increases in each group. To investigate the results theoretically, the analysis based on Euler’s buckling theory and slenderness ratio is conducted, indicating that the theory is only valid for the high-AR group herein. Besides, considering the correction factor, *P_cr_* agrees well with the experimental results.

## 1. Introduction

Membranes with microdot apertures are widely used in lithography masks, etching masks, microfluidic devices, membranes, and filters [1,2,3]. Based on the membrane characteristics such as mechanical rigidity, surface characteristics, and optical transparency, various materials have been used for fabricating membranes as summarized in Table S1 [4,5,6]. Small-sized silicon nitride (SiNx) membranes with precisely controlled apertures on rigid silicon (Si) support layer, which is fabricated by a conventional lithography process, have been used in various fields requiring mechanical rigidity [7]. However, silicon-based membranes are vulnerable to external forces, and it is challenging to apply such membranes to curved surfaces due to their high rigidity. Besides, the manufacturing process is complicated and requires high cost, even for small-area manufacturing. To overcome these issues, researches on fabricating polymeric membranes have been conducted, which is advantageous for easy processability, flexibility, transparency, and low cost [8]. Among many types of polymeric membrane, UV-cured membranes using the oxygen-scavenging effect of the UV curable materials have been reported [9]. To fabricate polymeric membranes with microdot apertures, UV-curable prepolymer was dispensed on the patterned polydimethylsiloxane (PDMS) mold, and the flat PDMS mold was put on it. After contacting each layer, the appropriate vertical pressure was applied to the assembly and followed by the UV curing process. This simple and rapid process is advantageous for time-saving, low cost, scalability, and potential to be used in automated large-area roll-to-roll processes. However, due to the inherently deformable characteristic of PDMS, including buckling, bending, clustering, and fracture in response to the external load, it is still challenging to construct upstanding microdot apertures without clogging or bending over the whole area of UV-cured membrane through this process. As the structural aspect ratio (AR: height-to-diameter (*H*/*D*) ratio) increases, the deformation of the structures is inevitable, which leads to the process failure of the membrane with apertures. To achieve reliability and reproducibility of the process and advance toward realization of large-scale fabrication and automatization, this issue should be addressed. Since previous studies did not address the processibility depending on the structural features and vertical pressure, process criteria considering the AR and vertical pressure should be developed. Here, we have established a design rule based on critical buckling pressure (*P_cr_*) to prevent the buckling phenomenon. The experiments were conducted using different structures with various ARs of 1.6, 2.0, 4.0, and 5.33. Moreover, additional structures with different diameters and heights of the pillars with same ARs of 2.0 and 4.0 were prepared to verify whether the AR is the only determining factor for structural stability in the structural features.

## 2. Materials and Methods

### 2.1. Materials

Commercially available UV curable polyurethane acrylate (PUA) resin (MINS-311RM, Changsung Sheet, Cheonan, Korea) was used in the experiment. The PUA prepolymer resin consists of a photoinitiator, a UV curable releasing agent, and a prepolymer for crosslinking with acrylate functional groups.

### 2.2. Preparation of Polydimethylsiloxane (PDMS) Patterned Array Mold

To fabricate the patterned polydimethylsiloxane (PDMS) molds, PDMS was poured into the hole patterned silicon masters, which were made by photolithography and reactive ion etching. The surface of the silicon masters was treated with C_4_F_8_ gas to detach the patterned PDMS polymer easily. Next, the mixture of PDMS (Sylgard 184 PDMS elastomer, Dupont, Wilmington, DE, USA) base and curing agent with a weight ratio of 10:1 was poured on the patterned silicon masters and cured for 2 h at 70 °C. The cured PDMS molds were carefully peeled from the silicon masters [10].

### 2.3. Fabrication of Polymeric Stencil with Microdot Apertures

**Figure 1:**
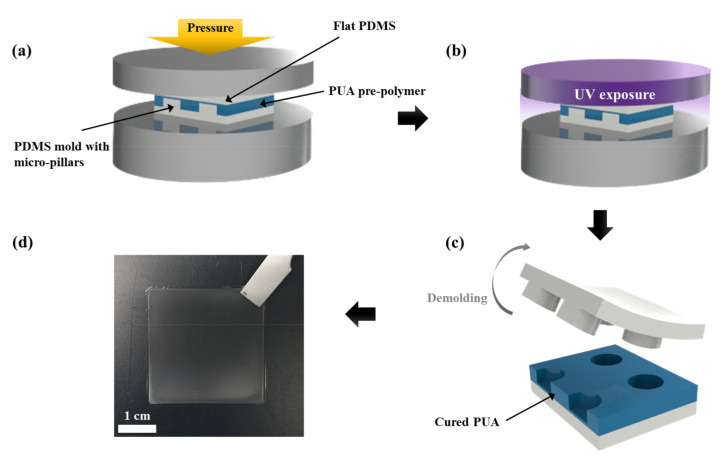
Schematic of the fabrication process of polymeric stencils with microdot apertures using UV imprint equipment. (**a**) Applying external pressure to the assembly of polydimethylsiloxane (PDMS) pillar patterned mold-polyurethane acrylate (PUA) prepolymer-PDMS flat mold, (**b**) ultraviolet (UV) irradiation process, (**c**) peeling off the PDMS molds, (**d**) Digital camera image of the free-standing PUA stencil with microdot apertures.

The fabrication process of the polymeric stencil with microdot apertures using a piece of imprint equipment is illustrated in Figure 1a–d. After dispensing the PUA resin onto micropillar patterned PDMS molds, a flat PDMS mold was placed on the PUA resin. The prepared sandwich-like assembly (patterned-PDMS-mold/PUA-resin/flat-PDMS-mold) were inserted into the UV imprint equipment and pressed through the imprint plate connected to the compressor line (Figure 1a). The applied pressure was regulated by adjusting the valve in the compressor. Next, the assembly was exposed to UV light with the intensity of 2 mWcm^−2^ (Figure 1b), measured using a UV light meter (UV-340A, Lutron Electronic, Coopersburg, PA, USA) for 10 s (Figure S1 shows the camera image of the equipment used in experiment). After turning off the UV light, releasing the assembly from the external pressure, and detaching the flat PDMS mold from the sandwiched layer, the cured PUA membrane was carefully detached from the micropillar patterned PDMS mold (Figure 1c) and then the free-standing PUA stencil with microdot apertures was successfully constructed (Figure 1d). In this process of Figure 1a,b, oxygen gas is infiltrated into the skin surface of the PUA prepolymer layers due to the gas permeability of PDMS, which is called the oxygen-infiltrated layer (OIL), as previously investigated. The UV curing in this layer is delayed from the existence of the oxygen gas, which is called the oxygen-scavenging effect. When the flat and patterned PDMS surfaces get close enough to make contact, the overlapped OILs further delay the UV curing, leading to the formation of the apertures in the polymeric stencil structures [9]. Finally, the constructed PUA membranes were exposed to UV light without any external load in the UV imprint equipment for more than 6 h to fully cure the uncured PUA resin on its surface.

### 2.4. Physical Analysis

Scanning electron microscopy (SEM) images were obtained using field emission SEM (FE-SEM, JSM-7800F, JEOL, Tokyo, Japan) with 10.0-kV acceleration voltage. Also, optical microscope (OM) images were obtained by optical microscopy (BX53M, Olympus, Tokyo, Japan). To confirm the shape of the apertures for each case, the constructed polymeric stencils were prepared by freeze-fracturing with liquid nitrogen and observed through SEM and OM images.

## 3. Results and Discussion

### 3.1. Fabrication of the Polymeric Stencil with Microdot Apertures with the Variation of Structural Aspect Ratio

Controlling the distance between the PDMS surfaces, which is adjusted by the externally applied pressure from the compressor in the system, is more important in the process of fabricating the polymeric stencil than applying pressure by hand to achieve reproducibility, reliability, uniformity, and accuracy. When the applied pressure is insufficient to secure the proper distance between the PDMS surfaces, the PUA layers, where the apertures should be formed, would be fully cured, resulting in the blocking of the apertures in the PUA stencil. However, if excessive pressure is applied, the pillars in the patterned PDMS in contact with the flat PDMS would be buckled or fractured, which is considered a process failure. The previous studies for fabricating a UV-cured membrane with apertures exhibit the need for investigation of properly applied pressure depending on the structural dimension as summarized in Table S2. Therefore, it is essential to establish a design rule for the *P_cr_* depending on the geometrical features of the pillars to prevent buckling-induced process failure. This approach would provide concrete fundamentals for the fabrication criteria progressed from an empirical approach. To investigate the structural stability, six patterned PDMS molds with micropillars having different *D* and *H* were set. The detailed information on the fabrication process of PDMS molds and patterned silicon masters is provided in the Section 2.2.

**Figure 2:**
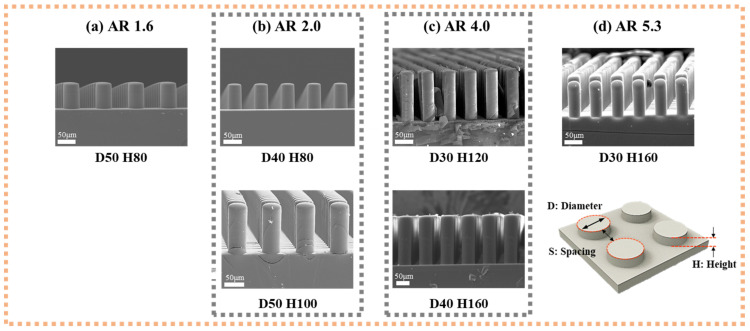
Scanning electron microscopy (SEM) images of prepared PDMS pillar patterned molds: (**a**) aspect ratio (AR) 1.6 [diameter (D) 50, height (H) 80], (**b**) AR 2.0 [D40 H80, D50 H100], (**c**) AR 4.0 [D30 H120, D40 H160], (**d**) AR 5.3 [D30 H160].

The scanning electron microscopy (SEM) images in Figure 2 show the geometry of each experimental sample. The samples have geometrical features as listed below: (1) *D* = 50 μm, *H* = 80 μm, and AR = 1.6; (2) *D* = 40 μm, *H* = 80 μm, and AR = 2.0; (3) *D* = 50 μm, *H* = 100 μm, and AR = 2.0; (4) *D* = 30 μm, *H* = 120 μm, and AR = 4.0; (5) *D* = 40 μm, *H* = 160 μm, and AR = 4.0; (6) *D* = 30 μm, *H* = 160 μm, and AR = 5.3. The samples were mainly categorized into four kinds of AR (1.6, 2.0, 4.0, and 5.3). For the case of AR = 2.0 and 4.0, the samples with different *D* and *H* while fixing the AR were prepared to confirm if AR is the only factor in geometry, which determine the mechanical stability herein. With the prepared patterned PDMS mold, the fabrication of the polymeric stencil with microdot apertures was conducted with the sandwich-like assembly as mentioned above. The applied vertical pressure to the assembly was increased gradually and discretely through the imprint machine. For each case, the applied imprint force was converted into the pressure per single pillar. The pressure that induces the buckling phenomenon is denoted as the experimental buckling pressure.

### 3.2. Buckling Phenomena of the Microdot Apertures in Stencils Depending on Aspect Ratio

**Figure 3:**
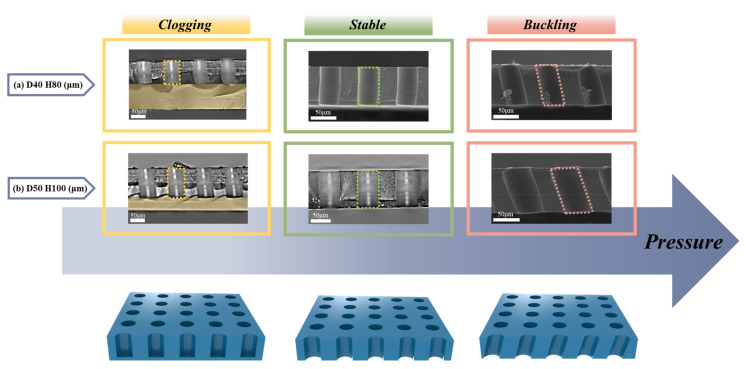
(**a**,**b**) SEM and optical microscopy (OM) images of constructed polymeric stencil membranes with aspect ratio of 2 and imprint pressure variation. Structural geometry with a diameter of 40 μm and a height of 80 μm (**a**), and a diameter of 50 μm and a height of 100 μm (**b**).

First, to confirm if the other geometrical features, such as *D* and *H*, affect the buckling phenomena in the same AR condition, the experiment was performed using two pillar structures with same AR = 2.0 (Figure 3). The results show that the shape of the aperture in the polymeric stencils, depending on the applied pressure, can be categorized into three cases: (1) clogging, (2) stable, and (3) buckled. Without applying external pressure to the assembly, the apertures in the PUA stencil are blocked, which is called clogging. If the appropriate pressure, which is 0.091 MPa to the single pillar in this case, is applied to the assembly, the upstanding apertures without slanting and bending in the PUA stencil are constructed stably. Also, when the pressure over 0.17 MPa is applied, the apertures in the PUA stencil are slanted, which is a buckling phenomenon. This pressure is denoted as *P_cr_*. From the summarized results in Table 1, the pressures that transit the state of the apertures from stable to buckled were comparable even with different *D* and *H* of the pillars, indicating that the critical factor for buckling is the AR, not *D* or *H*.

**Figure 4:**
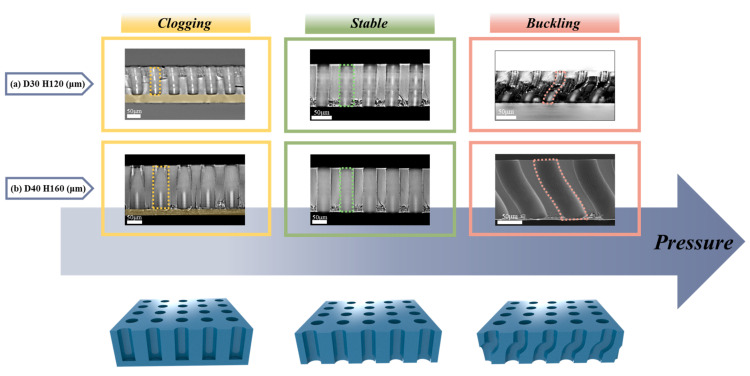
(**a**,**b**) SEM and OM images of constructed polymeric stencil membranes with aspect ratio of 4 and imprint pressure variation. Structural geometry with a diameter of 30 μm and a height of 120 μm (**a**), and a diameter of 40 μm and a height of 160 μm (**b**).

**Figure 5:**
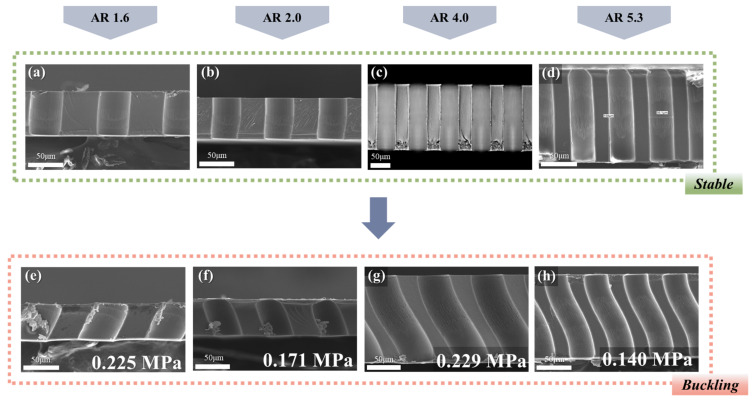
(**a**–**h**) SEM and OM images of constructed polymeric stencil membranes with varying aspect ratios. Polymeric stencil membranes in stable state (**a**–**d**), and in buckled state with structural AR of 1.6, 2.0, 4.0, and 5.3 (**e**–**h**).

The same trend is observed in the other case, as displayed in Figure 4 and Table 2. The experiments using two different samples with same AR = 4.0 were conducted, and the structural features depending on the pressure are also classified as clogging, stable, and buckled, which is the same with the previous result. The *P_cr_* of the two samples was the same, with a value of ~0.229 MPa for both. From the results, it is also confirmed that the only geometric factor affecting the buckling phenomenon is AR. Based on this fact, structures with four ARs (1.6, 2.0, 4.0, and 5.3) were prepared, and the stencils were fabricated with each structure (Figure 5). The pressures at the transition from the stable to the buckled state, which are *P_cr_*, were 0.225 MPa (AR = 1.6), 0.171 MPa (AR = 2.0), 0.229 MPa (AR = 4.0), and 0.140 MPa (AR = 5.3) (Table 3). Note that the shapes in the buckling state appear differently depending on the AR; the structures can be classified according to the buckled shapes into two groups: low (AR = 1.6 and 2.0) and high (AR = 4.0 and 5.3) AR groups. The structures with relatively low AR were slanted without a curved wall and point of inflection, while those with relatively high AR were bent with a curved wall. Obviously, *P_cr_* decreases in each group as the AR increases since the slender and slim structures would be easily deformed and unstable. However, when comparing the results of the high AR group and low AR group, the critical buckling pressure is expected to be much larger in the case of the low AR group compared to the high AR group, however, experimental results were not. The overall experimental pressures at which the membranes are in stable and buckling states are plotted in Figure S2. To investigate the previous experimental results theoretically, the comparative analysis of *P_cr_* based on the commonly known Euler’s buckling theory was conducted. Two main issues were encountered in the analysis—first, a comparison of the theoretical and experimentally obtained values of *P_cr_*, and second, the criteria for classification into low or high AR.

### 3.3. Theoretical Approach to Determine the Critical Buckling Pressure

**Figure 6:**
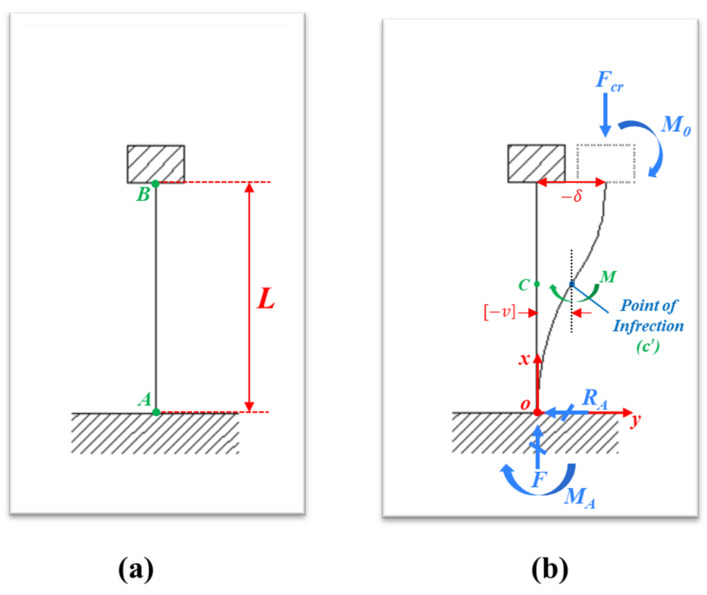
A simple analytical schematic of a pillar in (**a**) stable and (**b**) buckled states.

To address both issues, the proposed simple analytical model is illustrated in Figure 6. To calculate the theoretical value of *P_cr_*, the end conditions of the pillar in the model should be set reflecting the behavior of the experimentally used PDMS pillar. In this analytical model, the bottom and the vertical line connected as one body represents the patterned PDMS, while the top cover represents the flat PDMS. The bottom-end condition of the pillar is fixed at the base, and the upper-end condition of the pillar is assumed to be rotation-fixed and translation-free. Figure 6a indicates the state of the pillar with a height of *L* without an applied load. Figure 6b demonstrates the buckling shape of the pillar when the critical buckling load (*F_cr_*) is applied to the pillar, which corresponds to the shape of the apertures in Figure 5g,e. In the schematic model, the point of injection (*c*′) is defined as the center point of the deflected pillar where the bending moment is zero. The theoretical *F_cr_* can be obtained by solving the bending-moment equation considering the zero-moment at the point of injection (*c*′) in Equation (1), which is called Euler’s buckling equation (see detailed derivation for *F_cr_* in Equation S1 from Euler’s Buckling Equation).

(1) $$F_{cr}=\frac{\pi^{2}E^{*}I}{L^{2}}\;\left[N\right]$$

*P_cr_* can be calculated by dividing *F_cr_* by the top surface area of the pillar of the patterned PDMS mold as expressed in Equation (2). The *P_cr_* in this equation corresponds to the pressure applied to a single pillar among numerous pillars in the patterned PDMS mold with an area of 9 cm^2^.

(2) $$P_{cr}=\frac{F_{cr}}{A}=\frac{\pi E^{*}}{16}\times {\left(\frac{D}{L}\right)}^{2}=\frac{\pi E^{*}}{16}\times {\left(\frac{1}{AR}\right)}^{2}\;\left[N\;m^{-2}\right]$$

Here, *I* is the moment of inertia with respect to the principal axis of buckling, which is $\frac{\pi D^{4}}{64}$, *A* is the top surface area of the pillar, which is $\frac{\pi D^{2}}{4}$, and the *L* is the height of the pillar (*H*). E^*^ = *E*/(1 − *v*^2^), where *v* is the Poisson’s ratio and *E* is the Young’s modulus. E^*^ in Equations (1) and (2) is the combined elastic modulus, which is commonly used for large deformations considering the material’s Poisson’s ratio [11,12,13]. After substituting the Poisson’s ratio of PDMS as 0.5, E^*^ is expressed as 4*E*/3 [14,15].

To elucidate the applicability of this equation to the cases in this study, the criteria for applying Euler’s buckling theory were investigated. Since Euler’s buckling equation is derived assuming that the pillars under the external load are extremely thin like a thread or string, it is not that suitable to assume such for the pillars used herein. In particular, the low-AR structures are likely to be excluded from the applicable range of Euler’s buckling equation. To set the criteria for the applicability of Euler’s buckling equation, the slenderness ratio was introduced and used, which is defined as the ratio of the length of the structure to its least radius of gyration *r*. Based on the definition, the slenderness ratio can be expressed as follows:

(3) Lr=LIA=Lπd464∗4πd2=Ld4=4Ld=4AR,

where the radius of gyration (*r*) describes the cross-sectional area distribution in a pillar around its centroidal axis, which is $\sqrt{\frac{I}{A}}$. This result indicates that the slenderness ratio equals four fold of the AR. The critical slenderness ratio (*L*/*r*)*_c_*, which determines the applicability of Euler’s buckling equation, is obtained by assuming that the material’s critical stress ($\sigma_{cr}$) is equal to its proportional limit ($\sigma_{pl}$) [16]. Euler’s buckling equation is applicable in the range above the critical slenderness ratio; however, it is not applicable in the range below the critical slenderness ratio. Determining the critical slenderness ratio requires calculating $\sigma_{cr}$ and $\sigma_{pl}$. The critical stress ($\sigma_{cr}$) can be expressed by dividing the critical load by the top surface area of the single pillar (i.e., equal to the *P_cr_*), and the relationship between $\sigma_{cr}$ and (*L*/*r*)_*c*_ can be re-expressed by substituting Equation (2) into Equation (3) as follows:

(4) $$\sigma_{cr}=P_{cr}=\frac{F_{cr}}{A}=\frac{\pi^{2}E^{*}}{{\left(L/r\right)}_{c}{}^{2}}\;\left[N\;m^{-2}\right]$$

After rearranging the equation for (*L*/*r*)*_c_* and assuming that $\sigma_{cr}$ is the same as $\sigma_{pl}$, the equation is expressed as follows:

(5) $${\left(\frac{L}{r}\right)}_{c}=\sqrt{\frac{\pi^{2}E^{*}}{\sigma_{pl}}}=\sqrt{\frac{2\pi^{2}E^{*}}{\sigma_{y}}}$$

**Figure 7:**
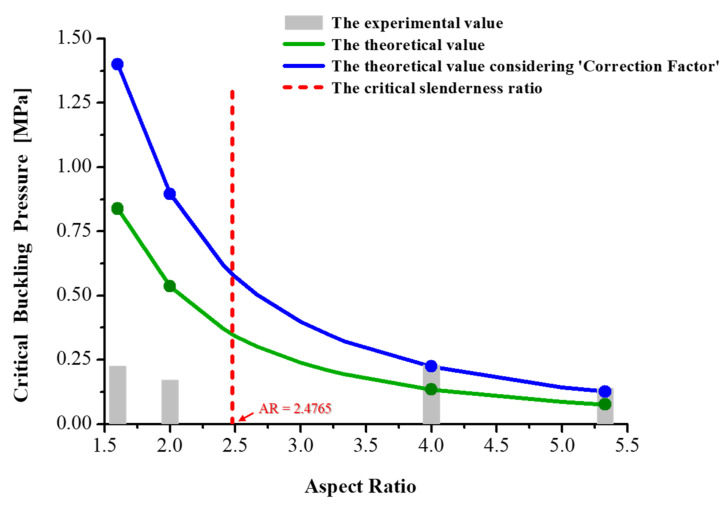
Plot of experimentally obtained and theoretically calculated critical buckling pressures of pillared structures as a function of aspect ratio: (gray bar) is the experimental values, (green line) is the theoretical values, (blue line) is the correction factor calibrated theoretical values, and (red dotted line) indicates the criteria of applicability of Euler’s buckling theory.

In Equation (5), $\sigma_{pl}$ is generally considered half of the material’s yield strength, $\sigma_{y}$ [16,17,18,19]. In a previous study, the yield strength of PDMS was reported as 700 kPa [20]. E^*^ is calculated as 3.48 MPa using the *E*-value of PDMS (2.61 MPa) [21]. Based on the information, (*L*/*r*)*_c_* was calculated as 9.906. By considering the relationship between the critical slenderness ratio and AR in Equation (3), the critical AR (*AR_c_*) as an indicator of applicability of Euler’s buckling equation was calculated as 2.4765. Thus, Euler’s buckling equation is only valid for the pillar structure with AR over ~2.5 in the case of using PDMS as molds. From the results, the calculated critical slenderness ratio, the theoretical *P_cr_*, and the experimentally obtained buckling pressure are plotted in Figure 7. As shown in the graph, in the applicable range of Euler’s buckling equation, the theoretical (green line) and the experimental (gray bar) values are incompletely matched, indicating that despite applying higher pressure than *P_cr_*, the polymeric stencil was stably made without the buckling phenomenon. This result can be attributed to the inaccuracy in the following cases, which is not considered while analyzing the buckling phenomenon using Euler’s buckling equation [22,23]:

- Inhomogeneity due to the inherent non-uniformity of the material and imperfect mixing process of PDMS base and curing agent of PDMS.
- The imperfect flatness of the UV-imprinting machine used in the experiment.
- Slightly different distances from the substrate to the top surface of each pillar due to the finely inclined PDMS mold.

**Figure 8:**
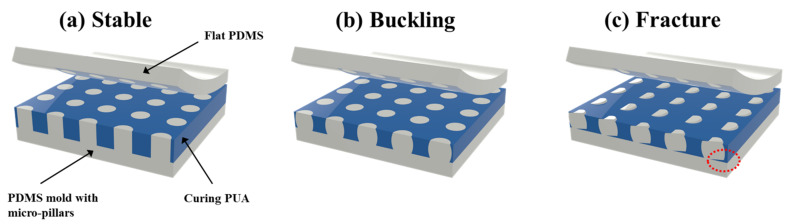
(**a**–**c**) Schematic of the ideal shape of pillars with low aspect ratio with variations of applied pressure. Stable (**a**), buckled (**b**), and fractured states (**c**).

To correct the inaccuracy, the correction factor was considered, the value of which is 1.67 as derived from the comparison between the experimental and theoretical results. Consequently, in the applicable range of Euler’s buckling equation in Figure 7, which are the cases of AR = 4.0 and 5.3, the experimental results (Gray bar) and the correction-factor-calibrated theoretical values (blue line) fit well. However, in the non-applicable range of Euler’s buckling equation, which are the cases of AR = 1.6 and 2.0, the experimentally obtained values are more than thrice lower the theoretical values, even when the correction factor is considered. This result indicates that the experiment-based approach should be used to anticipate the structural deformation of the structures with low AR below *AR_c_* due to the mismatch of the experimental result and Euler’s buckling theorem. Apparently, the resultant shapes of the low-AR structures were slanted without the curved wall, whereas the high-AR structures were bent with a curved wall. Also, the lateral shift of the pillar top surface of the low-AR structures was more alleviated than that of the high-AR structures under the comparable applied pressure. This trend implies that although the deformation of the low-AR structures occurs below the anticipated theoretical buckling pressure, the amount of deformation was restrained compared to the high-AR structures. It is well known that the large deformation for a short pillar is restrained, and since the structural stress is accumulated due to less deformation, it would be fractured rather than deformed. In this aspect, ideally, the low-AR structures under compression are likely to be expanded laterally (Figure 8), which is slightly different from the resultant shape based on observation. This trend is ascribed to the inevitably and unintentionally applied shear stress from the process inaccuracy, including the material’s inhomogeneity and imperfect flatness. Hence, to manufacture completely upstanding apertures without deformation in the polymeric stencil, the design rules should be considered as follows. First, in the case of the pillars with higher AR than AR_c_, the pressure on a single pillar should be lower than the predicted *P_cr_* based on Euler’s buckling theory. Meanwhile, in the case of the pillars with lower AR than AR_c_, an experimental approach to obtain *P_cr_* is required rather than the theoretical approach based on Euler’s buckling equation. Second, in both cases, it is extremely important to minimize the aforementioned inaccuracy, including material inhomogeneity and imperfect flatness in the process.

## 4. Conclusions

We fabricated UV-cured polymeric stencils with microdot apertures having varied AR of the structures based on the scavenging effect of PDMS molds. In the fabrication process, the buckling phenomenon of the PDMS pillars was observed when the applied vertical pressure increased above a certain pressure, which is *P_cr_*. As a result of the investigation of the buckling behavior of the PDMS pillar during the process, the structures with the same AR showed almost the same *P_cr_*, which confirms that the structural factor determining the buckling phenomenon of the single pillar is AR, not *D* or *H*. Furthermore, based on the observation of the buckled morphology, the structures can be classified into two groups: a low-AR group (AR = 1.6 and 2.0), and a high-AR group (AR = 4.0 and 5.3). In each group, *P_cr_* decreases as the AR increases, as expected. Also, the comparative analysis of *P_cr_* based on Euler’s buckling theory was conducted to validate the buckling behavior. By considering the slenderness ratio, which is calculated as four times the AR in this study, we found the applicable range of Euler’s buckling theory. *AR_c_* was determined as 2.476; therefore, the analysis based on Euler’s buckling theory for the high-AR group is only valid herein. By considering a correction factor of 1.67, the experimentally obtained values and the calibrated theoretical buckling pressure agreed well for the high-AR group, but not for the low-AR group, in which the experimental approach rather than the theoretical approach should be considered. Such a design rule for preventing the buckling phenomenon can be used as an indicator to produce stable polymeric stencils with microdot apertures and can be advantageously applied to scalable processes, including the roll-to-roll process for manufacturing polymeric membranes for applications in biomedical, microfluidic, and flexible devices.

## Figures and Tables

**Table 1:** Applied imprint pressure during fabrication of polymeric stencil membranes with aspect ratio of 2.

Sample/Case	Clogging (MPa)	Stable (MPa)	Buckling (MPa)
**H80 D40 (μm)**	0	0.091	0.171
**H100 D50 (μm)**	0	0.091	0.175

**Table 2:** Applied imprint pressure during fabrication of polymeric stencil membranes with aspect ratio of 4.

Sample/Case	Clogging (MPa)	Stable (MPa)	Buckling (MPa)
**H120 D30 (μm)**	0	0.175	0.229
**H160 D40 (μm)**	0	0.168	0.229

**Table 3:** Applied pressure (buckling phenomenon inclusive) depending on aspect ratio.

Aspect Ratio	1.6	2.0	4.0	5.3
**Pressure (MPa)**	0.225	0.171	0.229	0.140

## Data Availability

Not applicable.

## Supplementary Materials

The following are available online at https://www.mdpi.com/article/10.3390/polym13244361/s1, Figure S1: Camera images of UV-imprinting equipment, chamber interior, and UV exposure process with pressure application, Equation S1. Derivation of critical buckling load (*F_cr_*) from Euler’s Buckling Equation, Figure S2: A plot of applied pressure with variations of aspect ratios, which indicates the pressure for the stable state and the buckling state, Table S1: Summarized processes for fabricating membranes with nano/micro apertures, Table S2: Summarized process condition of geometrical dimension and applied pressure to fabricate the UV cured polymeric membrane with apertures. Supplementary data to this article can be found in the online version or from the author.
