# Supplementary material for: Design Rule for Constructing Buckling-Free Polymeric Stencil with Microdot Apertures
Source: Polymers (Basel). 2021 Dec 13;13(24):4361. doi: 10.3390/polym13244361 (PMC8709208; doi:10.3390/polym13244361)
Supplement: Supplementary file 1 [file polymers-13-04361-s001.zip › polymers-1473692-supplementary.pdf]

polymers

MDPI

Supplementary Materials

Design Rule for Constructing Buckling-Free Polymeric Stencil with Microdot Apertures

Minju Kim [1,†], Jinwon Lee [1,†], Junsoo Kim [2], Segeun Jang [3] and Sang Moon Kim [1,*]

1 Department of Mechanical Engineering, Incheon National University, Incheon 22012, Korea; 202121050@inu.ac.kr (M.K.); iljwl92@gmail.com (J.L.)
2 John A. Paulson School of Engineering and Applied Science, Harvard University, Cambridge, MA 02138, USA; junsookim@g.harvard.edu
3 School of Mechanical Engineering, Kookmin University, Seoul 02707, Korea; sjang@kookmin.ac.kr
* Correspondence: ksm7852@inu.ac.kr
† Contributed equally as first authors.

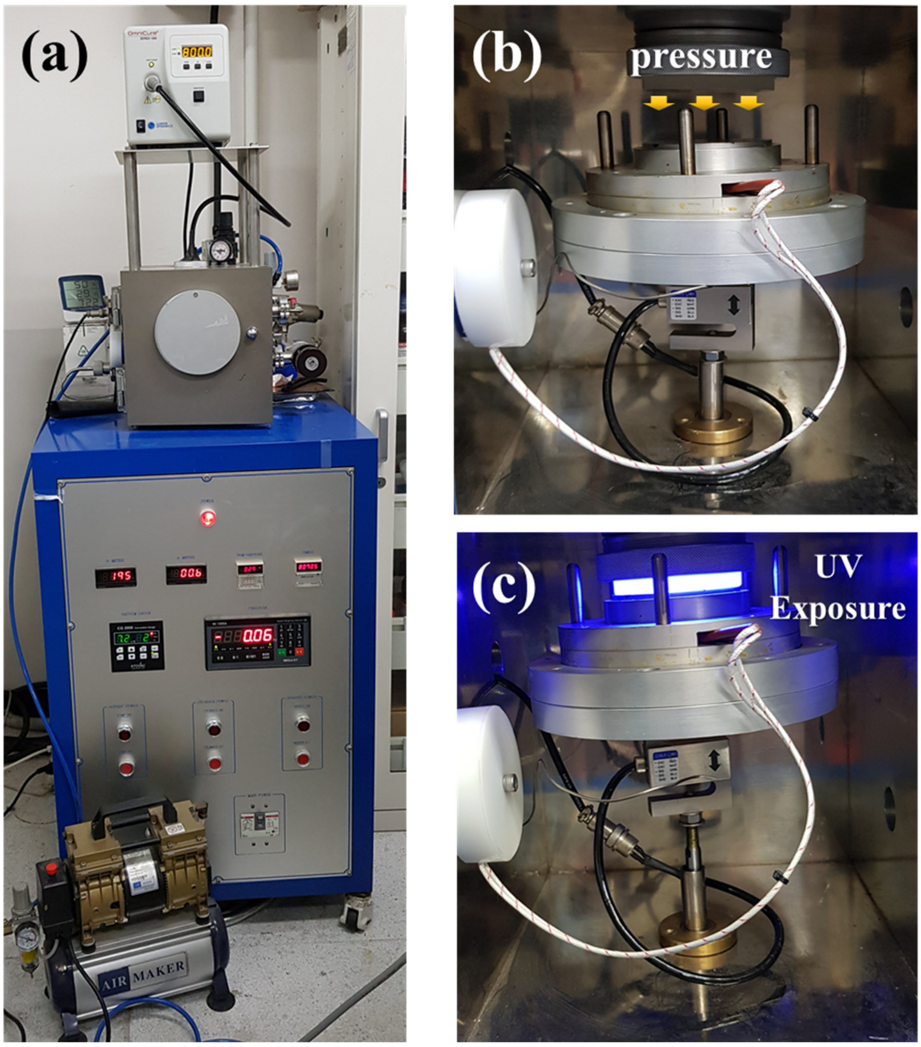

Figure S1. Camera images of (a) UV-imprinting equipment, (b) chamber interior, and (c) UV exposure process with pressure application.

Equation S1. Derivation of critical buckling load (F_{cr}) from Euler's Buckling Equation.

The bending moment at distance x from the base is given as $M = -M_0 - F(-\delta + v)$. The differential equation of the deflection curve is given as

$$EIv'' = M = -M_0 - F(-\delta + v) \quad (1)$$

$$v'' = -\frac{M_0}{EI} - \frac{F}{EI}(-\delta + v) \quad (2)$$

where I is the moment of inertia, E is the Young's modulus, M is the bending moment, v is the deflection in the y-direction, F is the vertical load, and δ is the deflection at the upper end of the pillar. After assuming $\frac{F}{EI} = k^2$, Equation 1 is expressed as follows

$$v'' + k^2 v = k^2\left(\delta - \frac{M_0}{F}\right) \quad (3)$$

which is a second-order differential equation with constant coefficients (δ). The general solution consists of homogenous and particular solutions. First, in the case of the homogeneous solution, which is the solution obtained by replacing the right-hand side with zero, expressed as $v'' + k^2 v = 0$. Then, the homogeneous solution is derived as

$$v_H = C_1 coskx + C_2 sinkx \quad (4)$$

Second, the particular solution is calculated by substituting v_P into the left-hand side of the differential equation. Then, the particular solution is expressed as

$$v_P = \delta - \frac{M_0}{F} \quad (5)$$

Consequently, the general solution of the equation is equal to the sum of v_H and v_P as follows.

$$v = C_1 coskx + C_2 sinkx + \left(\delta - \frac{M_0}{F}\right) \quad (6)$$

$$v' = -C_1 sinkx + C_2 coskx \quad (7)$$

Considering the boundary conditions, the deflection and slope are each equal to zero when $x = 0$. Therefore, $v(0) = 0$ and $v'(0) = 0$ are used to obtain the following results.

$$C_1 = \frac{M_0}{F} - \delta \text{ and } C_2 = 0 \quad (8)$$

Hence, Equation 2 is represented by $v = \left(\delta - \frac{M_0}{F}\right)(1 - coskx)$. Additionally, by substituting the last boundary condition, $v'(L) = 0$, which indicates that the slope is zero at the upper end of the pillar, $v'(L) = \left(\delta - \frac{M_0}{F}\right) k\, sin\, kL = 0$ is obtained.

It follows from this equation that either $\delta - \frac{M_0}{F} = 0$ or $sin\, kL = 0$. As a result, the only possibility for solving the equation is $sin\, kL = 0$. The equation $sin\, kL = 0$ is satisfied when

$$kL = n\pi \ (n = 1,2,3, \dots) \quad (9)$$

Finally, by using the expression $\frac{F}{EI} = k^2$ to obtain the following equation for Euler's critical buckling load, $F = \frac{n^2\pi^2 EI}{L^2}$ is obtained. Besides, the lowest critical buckling load is obtained by considering $n = 1$, and that is expressed as $F_{cr} = \frac{\pi^2 EI}{L^2}$ [N].

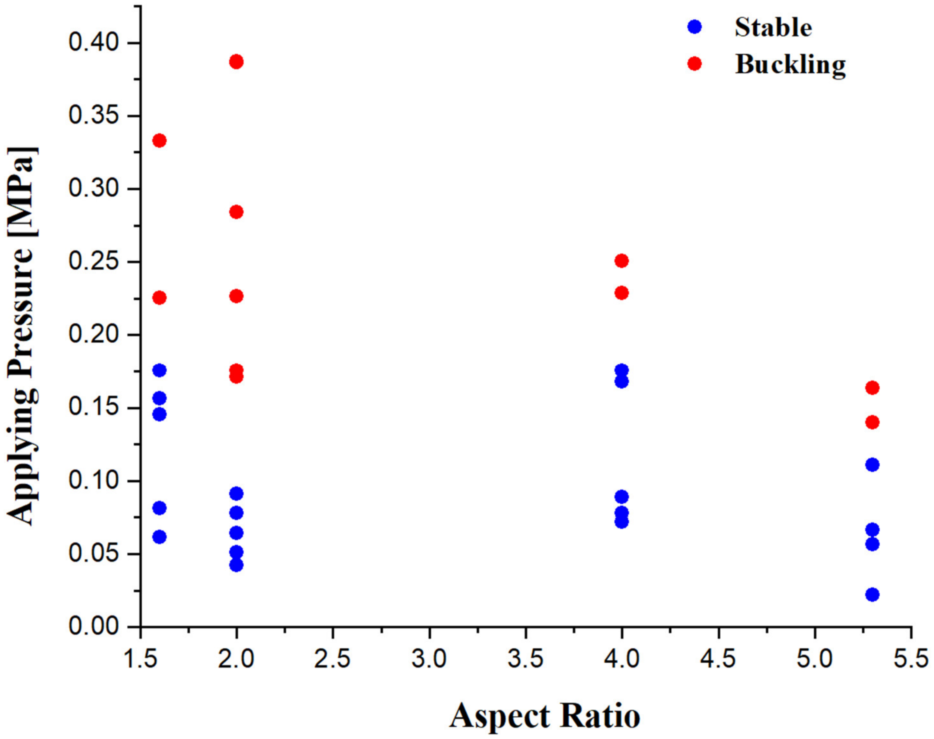

Figure S2. A plot of applied pressure with variations of aspect ratios. Blue dots indicate the pressure for the stable state, and red dots indicate the pressure for the buckling state.

Table S1. Summarized processes for fabricating membranes with nano/micro apertures.

Fabrication process	Pore size	Materials	Advantages	Limitation	Applications	References
Our process	800 nm - 500 µm	PUA, PFPE, NOA	Wide pore range, narrow pore size distribution, regularity, scalability, short fabrication time	Buckling phenomenon, material selectivity, compatibility with mold	Can be used for metal deposition mask, Water filtering	-
Electrospinning	500 nm - 150 µm	Gelatin, PVDF, PU, PA-6	Low cost, porosity controllability	non-uniform pores, low reproducibility	Tissue engineering/Water treatment/Drug delivery	[1]-[4]
Block copolymer templating	7 nm – 55 nm	NBCB-b-NBPLA, PS-b-PLA	Precision control, Reproducibility	Narrow pore size range, time-consuming, high cost	Bioseparation	[5]-[8]
Track etching	30 nm – 3 µm	PET, PC, PVDF	Narrow pore size distribution, Regularity	Limited geometry and scalability	Ionic transport/Bioseparation	[9]-[14]

References

1. [1] KI, Chang Seok, et al. Characterization of gelatin nanofiber prepared from gelatin–formic acid solution. *Polymer*, 2005, 46.14: 5094-5102.
2. [2] MIT-UPPATHAM, Chidchanok; NITHITANAKUL, Manit; SUPAPHOL, Pitt. Ultrafine electrospun polyamide-6 fibers: effect of solution conditions on morphology and average fiber diameter. *Macromolecular Chemistry and Physics*, 2004, 205.17: 2327-2338.
3. [3] BAE, Jiyeol; BAEK, Inchan; CHOI, Heechul. Efficacy of piezoelectric electrospun nanofiber membrane for water treatment. *Chemical Engineering Journal*, 2017, 307: 670-678.

4. [4] GABRIEL, Laís P., et al. Electrospun polyurethane membranes for Tissue Engineering applications. *Materials Science and Engineering: C*, 2017, 72: 113-117.
5. [5] GOPINADHAN, Manesh, et al. Thermally switchable aligned nanopores by magnetic-field directed self-assembly of block copolymers. *Advanced Materials*, 2014, 26.30: 5148-5154.
6. [6] PHILLIP, William A., et al. Self-assembled block copolymer thin films as water filtration membranes. *ACS applied materials & interfaces*, 2010, 2.3: 847-853.
7. [7] VAYER, Marylène, et al. Perpendicular orientation of cylindrical domains upon solvent annealing thin films of polystyrene-b-polylactide. *Thin Solid Films*, 2010, 518.14: 3710-3715.
8. [8] OLAYO-VALLES, Roberto, et al. Perpendicular domain orientation in thin films of polystyrene− polylactide diblock copolymers. *Macromolecules*, 2005, 38.24: 10101-10108.
9. [9] YAMEEN, Basit, et al. Synthetic proton-gated ion channels via single solid-state nanochannels modified with responsive polymer brushes. *Nano letters*, 2009, 9.7: 2788-2793.
10. [10] LIN, Lei, et al. Sensitive nanochannel biosensor for T4 polynucleotide kinase activity and inhibition detection. *Analytical chemistry*, 2013, 85.1: 334-340.
11. [11] DELAVARI, Armin, et al. Latex particle rejections from virgin and mixed charged surface polycarbonate track etched membranes. *Journal of Membrane Science*, 2019, 584: 110-119.
12. [12] MARTÍNEZ-PÉREZ, Paula; GARCÍA-RUPÉREZ, Jaime. Commercial polycarbonate track-etched membranes as substrates for low-cost optical sensors. *Beilstein journal of nanotechnology*, 2019, 10.1: 677-683.
13. [13] CUSCITO, Olivia, et al. Nanoporous β-PVDF membranes with selectively functionalized pores. *Nuclear Instruments and Methods in Physics Research Section B: Beam Interactions with Materials and Atoms*, 2007, 265.1: 309-313.
14. [14] ZHAO, C.-D.; VATER, P.; BRANDT, R. Further studies on the production of pvdf nuclear track microfilters. *International Journal of Radiation Applications and Instrumentation. Part D. Nuclear Tracks and Radiation Measurements*, 1991, 19.1-4: 829-834.

Table S2. Summarized process condition of geometrical dimension and applied pressure to fabricate the UV cured polymeric membrane with apertures .

Ref #	The method for applying pressure	Applied pressure	Mold-Material	Diameter	Height	Aspect ratio	Stability
Our results	UV imprint machine	1.75 bar<br>2.29 bar	PDMS-PUA	30 μm<br>30 μm<br>30 μm	120 μm<br>120 μm<br>120 μm	4<br>4<br>4	Stable<br>Buckled
[1]	Pressure by hand	~ 0.5 bar	PFPE-PUA	500 μm<br>20 μm<br>800 nm	20 μm<br>5 μm<br>800 nm	0.04<br>0.25<br>1	Stable<br>Stable<br>Stable
[2]	UV imprint machine	0.02 ~ 0.8 bar	PFPE-PUA	800 nm<br>50 μm<br>20 μm<br>150 μm	800 nm<br>20 μm<br>20 μm<br>50 μm	1<br>0.4<br>1<br>0.33	Stable<br>Stable<br>Stable<br>Stable
[3]	UV imprint machine	0.02 ~ 0.8 bar	PDMS-PUA	800 nm<br>20 μm<br>500 μm	800 nm<br>20 μm<br>20 μm	1<br>1<br>0.04	Stable<br>Stable<br>Stable
[4]	External pressure	2 bar	PDMS-PUA	60 μm<br>50 μm	20 μm<br>30 μm	0.33<br>0.6	Stable<br>Deformed
[5]	Roll-to-roll machine	6 bar<br>10 bar	PDMS-PUA	50 μm<br>50 μm	150 μm<br>150 μm	3<br>3	Stable<br>Buckled

References

15. [1] KIM, Junsoo, et al. Nonlinear frameworks for reversible and pluripotent wetting on topographic surfaces. *Advanced Materials*, 2017, 29.7: 1605078.
16. [2] CHO, Hyesung, et al. Replication of flexible polymer membranes with geometry-controllable nano-apertures via a hierarchical mould-based dewetting. *Nature communications*, 2014, 5.1: 1-10.
17. [3] CHO, Hyesung, et al. Multiplex lithography for multilevel multiscale architectures and its application to polymer electrolyte membrane fuel cell. *Nature communications*, 2015, 6.1: 1-8.

18. [4] PARK, Tae Hwan; KIM, Ju-Hyung; SEO, Soonmin. Facile and Rapid Method for Fabricating Liquid Metal Electrodes with Highly Precise Patterns via One-Step Coating. *Advanced Functional Materials*, 2020, 30.35: 2003694.
19. [5] LEE, Jihoon, et al. Scalable fabrication of flexible microstencils by using sequentially induced dewetting phenomenon. *ACS omega*, 2017, 2.3: 1097-1103.
